# Matching Reads to Many Genomes with the *r*-Index

**DOI:** 10.1089/cmb.2019.0316

**Published:** 2020-04-08

**Authors:** Taher Mun, Alan Kuhnle, Christina Boucher, Travis Gagie, Ben Langmead, Giovanni Manzini

**Affiliations:** ^1^ Department of Computer Science, Johns Hopkins University, Baltimore, Maryland.; ^2^ Department of Computer and Information Science and Engineering, University of Florida, Gainesville, Florida.; ^3^ Faculty of Computer Science, Dalhousie University, Halifax, Canada Center for Biotechnology and Bioengineering, Santiago, Chile School of Computer Science and Telecommunications, Universidad Diego Portales, Santiago, Chile.; ^4^ Department of Science and Technological Innovation, University of Eastern Piedmont, Alessandria, Italy.

**Keywords:** Burrows–Wheeler Transform, indexing, *r*-index, pan-genomics

## Abstract

The *r*-index is a tool for compressed indexing of genomic databases for exact pattern matching, which can be used to completely align reads that perfectly match some part of a genome in the database or to find seeds for reads that do not. This article shows how to download and install the programs ri-buildfasta and ri-align; how to call ri-buildfasta on an FASTA file to build an *r*-index for that file; and how to query that index with ri-align.

## 1. Background

The Burrows–Wheeler Transform (BWT) (Burrows and Wheeler, 1994) and the Full-text Minute-space FM index (FM-index) (Ferragina and Manzini, 2005) are central to the most popular short-read aligners, such as Burrows-Wheeler Aligner (BWA) (Li and Durbin, 2009) and Bowtie (Langmead et al., 2009), but until recently it was not known how to apply these concepts effectively to whole genomic databases. Building on previous authors' work (Mäkinen et al., 2010; Gagie et al., 2018) described how a fully functional variant of the FM-index for such a database could be stored in reasonable space: their variant takes *O*(*r*) machine words, where *r* is the number of runs in the BWT of the database, and thus is called the *r-index*. Prezza gave a preliminary implementation, which was significantly extended by Boucher et al. (2019) and Kuhnle et al. (2019). This article is meant as a brief guide to the extended implementation. For help troubleshooting or to provide feedback, please submit an issue to our GitHub page, which also has more documentation.

## 2. Installation

In this section, we give installation instructions, assuming users are using Ubuntu with c++17-compliant compilers and are familiar with the Unix command line. If they are using another Unix-like system, then they should substitute the appropriate package manager for apt.

Users should first download some prerequisite packages, and the source code from the GitHub repository:

$ apt-get update

$ apt-get install -y build-essential cmake git python3 zlib1g-dev

$ git clone—recursive https://github.com/alshai/r-index

They should then compile the code, as follows; any missing dependencies will be automatically downloaded and compiled locally.

$ cd r-index

$ mkdir build

$ cd build

$ cmake..

$ make

$ make install

$ cd..

These commands will install the binaries ri-buildfasta and ri-align in the system's default bin location (e.g., /usr/local/bin for Ubuntu users), together with bigbwt (Boucher et al., 2019) and the SDSL library (Gog et al., 2014) (if it is not already present). If users want the binaries elsewhere, then they should use

$ cmake -DCMAKE_INSTALL_PREFIX = <dest>..

instead of cmake.. in the sequence of commands above, where <dest> is the desired destination directory.

## 3. Construction

Users can call ri-buildfasta to build the *r*-index for a collection of genomic sequences stored in an FASTA file, which can be gzipped. For example, to index the 2.7 MB file data/dengue/genome.fa.gz, which contains 2042 Dengue Type 1 genomes downloaded from the Virus Pathogen Database and Analysis Resource (ViPR) (Pickett et al., 2012) and occupies 22 MB uncompressed, they can use the following command:

$ ri-buildfasta -o dengue data/dengue/genome.fa.gz

This command saves an *r*-index as the two files dengue.ri, which contains the main data structures for the index, and dengue.1.ri, which contains mappings from offsets in the indexed text to the names of the sequences starting at those offsets. If users want the .ri files to have different names, they should change the -o parameter in the command. In this example, the .ri files have a total size of 2.4 MB, or 11% and 89% of the sizes of the indexed text when uncompressed and gzipped, respectively; once they have been built, data/dengue/genome.fa.gz is no longer necessary.

By default, ri-buildfasta uses bigbwt, which efficiently handles most large repetitive text collections. For very small or insufficiently repetitive collections, however, the sais algorithm (Nong et al., 2010) might be faster, so users can change the construction algorithm using the -b parameter, as follows:

$ ri-buildfasta -b sais -o dengue data/dengue/genome.fa.gz

On an Intel i7-7700HQ 2.80 GHz laptop, building the index for data/dengue/genome.fa.gz with bigbwt takes about 3 seconds and 47 MB of memory, while building it with sais takes about 10 seconds and 104 MB of memory. With bigbwt, we have built a 665 MB *r*-index for a collection of 2000 copies of human chromosome 19 from different individuals, which take 110 GB uncompressed, in under 5 hours on a server using 41 GB of RAM and one thread. At the time of writing, the use of multiple processors does not significantly reduce the construction time, but that should change in the near future.

## 4. Alignment

Users can call ri-align to search for a collection of patterns stored in an FASTQ file. For example, to use the *r*-index from the previous section to count the occurrences of the 1000 simulated 100-bp reads in the 219 KB file data/dengue/reads.fq, which were taken from the first genome in the dengue collection, they should use the following command:

$ ri-align count dengue data/dengue/reads.fq

Note that the index should be specified without the .ri extension. The output is piped to the standard output by default, but can of course be redirected to a file. If users actually want the positions of the reads' occurrences, they can call ri-align locate:

$ ri-align locate dengue data/dengue/reads.fq

By default, ri-align locate returns the positions of all the occurrences of each read in the indexed text. This can be very slow, especially if the read occurs many times, so users can include –max-hits <k> to limit the number of occurrences that are reported, where <k> is the desired number of occurrences:

$ ri-align –max-hits <k> locate dengue data/dengue/reads.fq

Users can include –max-range <k> to obtain the occurrences only of reads that occur at most <k> times:

$ ri-align –max-range <k> locate dengue data/dengue/reads.fq

On the same Intel i7-7700HQ 2.80 GHz laptop and with the output redirected to a file, counting the occurrences of each read takes a total of about 0.06 seconds and locating one occurrence of each read takes a total of about 0.1 second.

## 5. Interpreting Counts

The output of the ri-align count command consists of a series of lines with each one containing the read name; a fraction with the length of the longest suffix of the read that occurs in the database as the numerator, and the length of the read as the denominator; and the number of times it occurs. The fields are separated by tabs. For the example in the previous section, the output is as follows:

$ ri-align count dengue data/dengue/reads.fq

> simulated.0 100/100 22

> simulated.1 100/100 2

> simulated.2 100/100 2

> simulated.3 100/100 2

…

With some errors in the reads, the output changes as follows:

$ ri-align count dengue data/dengue/reads_w_errors.fq

> simulated.0.3edits 44/100 492

> simulated.1.2edits 78/100 1

> simulated.2.1edits 81/100 2

> simulated.3.0edits 100/100 2

…

This means, for example, that after we add errors to simulated.0 to create simlulated.0.3edits, the last 44 characters of the edited read occur as a substring 492 times in the the database, but the last 45 characters never occur as a substring.

## 6. Interpreting Locations

The output of the ri-align locate is in the SAM format (Li et al., 2009). The Sequence Alignment/Map (SAM) format begins with a line specifying the name and length of each sequence in the database (2042 for dengue), and follows with a line for each read consisting of the following tab-separated fields: read name, flag, reference name, position, MAPping Quality (MAPQ) score, CIGAR string, reference name for the next read in the read-pair, position of the next read in the read-pair, observed template length, read sequence, read quality scores, and miscellaneous tags.

The most important fields are the reference name, which identifies the sequence in the database containing the occurrence, and the position, which specifies that the location of the occurrence of the read is in that sequence. Since the *r*-index does not currently support mapping-quality calculation or read-pair/mate-pair alignment, the MAPQ field, the read-pair fields, and the observed template length field will all contain their respective “unknown”-type values. (For more details on the SAM format, see https://samtools.github.io/hts-specs/SAMv1.pdf).

For our running example without errors, the output (limited to one occurrence per read) is

$ ri-align –max-hits 1 locate dengue data/dengue/reads.fq

> @HD VN:1.6 SO:unknown

> @SQ SN:gb:KY474305|Organism:Dengue LN:10676

> @SQ SN:gb:JN638344|Organism:Dengue LN:10735

…

> @SQ SN:gb:AY835999|Organism:Dengue LN:10727

> simulated.0 0 gb:GQ868562|Organism:Dengue 2525 255 100M * 0 0

TGG…CTA ∼∼∼…∼∼∼ NH:i:22

> simulated.1 0 gb:KY474306|Organism:Dengue 10001 255 100M * 0 0

CAT…AGC ∼∼∼…∼∼∼ NH:i:2

> simulated.2 0 gb:KY474306|Organism:Dengue 8833 255 100M * 0 0

GAT…TTG ∼∼∼…∼∼∼ NH:i:2

> simulated.3 0 gb:KY474306|Organism:Dengue 8149 255 100M * 0 0

CTA…GAA ∼∼∼…∼∼∼ NH:i:2

…

with ellipses shortening the 100-character reads and strings of ∼ symbols.

At the moment, ri-align locate reports only on reads that match perfectly; of course, users have the option to use ri-align count to find the lengths of the longest suffixes that occur in the database, then search with ri-align locate only for those suffixes. This can be used to generate seeds for approximate pattern matching of reads with errors or unseen variations. Since Illumina reads tend to have more errors toward the end, users may want to truncate their reads to obtain longer matching suffixes.

## Availability

The source code for these programs is released under GPLv3 and available at https://github.com/alshai/r-index.
